# Glaucoma diagnostic performance of macular ganglion cell complex thickness using regular and long axial length normative databases

**DOI:** 10.1038/s41598-022-15255-x

**Published:** 2022-07-04

**Authors:** Henry Shen-Lih Chen, Xiao Chun Ling, Da-Wen Lu, Lan-Hsing Chuang, Wei-Wen Su, Yung-Sung Lee, Wei-Chi Wu, Po-Han Yeh

**Affiliations:** 1 Department of Ophthalmology, Chang Gung Memorial Hospital, No. 5, Fu‑Hsin Road, Linkou, Taoyuan, 333 Taiwan; 2 College of Medicine, Chang Gung University, Taoyuan, Taiwan; 3 Department of Ophthalmology, Tri-Service General Hospital, Taipei, Taiwan; 4 Department of Ophthalmology, Chang Gung Memorial Hospital, Keelung, Taiwan

**Keywords:** Eye diseases, Ocular hypertension, Optic nerve diseases

## Abstract

The risks of misdiagnosing a healthy individual as glaucomatous or vice versa may be high in a population with a large majority of highly myopic individuals, due to considerable morphologic variability in high myopic fundus. This study aims to compare the diagnostic ability of the regular and long axial length databases in the RS-3000 Advance SD-OCT (Nidek) device to correctly diagnose glaucoma with high myopia. Patients with high myopia (axial length ≥ 26.0 mm) in Chang Gung Memorial Hospital, Taiwan between 2015 and 2020 were included. Glaucoma was diagnosed based on glaucomatous discs, visual field defects and corresponding retinal nerve fiber layer defects. The sensitivity, specificity, diagnostic accuracy and likelihood ratios of diagnosing glaucoma via mGCC thickness in both superior/inferior and GChart mapping using the regular and long axial length normative databases. The specificity and diagnostic accuracy of mGCC thickness for distinguishing glaucomatous eyes from nonglaucomatous eyes among highly myopic eyes were significantly improved using the long axial length database (p = 0.046). There were also significant proportion changes in S/I mapping as well as GChart mapping (37.3% and 48.0%, respectively; p < 0.01) from abnormal to normal in the myopic normal eye group when using the long axial length normative database. The study revealed that clinicians could utilize a long axial length database to effectively decrease the number of false-positive diagnoses or to correctly identify highly myopic normal eyes misdiagnosed as glaucomatous eyes.

## Introduction

Glaucoma is regarded as a multifactorial optic neuropathy characterized by progressive loss of retinal ganglion cells, retinal nerve fiber layer (RNFL) thinning, and ultimately irreversible visual impairment. In East Asian countries, myopia affects a significant proportion of the population^1^. Studies have shown that moderate-to-high myopia is associated with increased risks of primary open angle glaucoma, ocular hypertension and normal tension glaucoma^2–4^. Similarly, a myopic fundus can pose significant challenges in correctly diagnosing glaucoma because of its considerable morphologic variability, tilted disc, physiologic cupping, shallower optic cups and peripapillary atrophy^5,6^. Thus, the risks of misdiagnosing a healthy individual as glaucomatous or vice versa may be high in a population with a large majority of highly myopic individuals.

Longer axial lengths (ALs) and vitreous chamber depths were also appreciated in myopic eyes^7^. The relationship of long AL and high myopia was first postulated by Von Graefe. Progression patterns in the topography of the posterior pole of highly myopic eyes, including concomitant decreased retinal thickness and macular atrophy, were associated with elongations of AL^8^. Consequently, regional variations in macular or RNFL thicknesses might affect the evaluation of glaucoma in highly myopic eyes^9^.

To resolve this issue, investigators have suggested evaluating assessments of macular ganglion cell complex (mGCC) thickness via spectral-domain optical coherence tomography (SD-OCT)^10,11^. The thickness of mGCCs can play an important role in the early diagnosis of glaucoma^12^. As a complementary measurement to RNFL thickness assessment, mGCC thickness assessment can aid in the clinical evaluation of glaucoma, even in highly myopic individuals^13,14^.

However, there are two aspects to be considered in the evaluation of thicknesses and significance maps of average mGCCs in highly myopic eyes. One is that the elongation of AL can produce ocular magnification of retinal images^15^. In instances where magnification is not corrected, the SD-OCT scan area will become wider in eyes with longer ALs, thus leading to possible misdiagnosis^16^. Another aspect to consider is that population data of individuals with high myopia, hyperopia or astigmatism are not usually included in the normative database of mGCC thicknesses^17,18^. This results in reduced accuracy and precision of glaucoma diagnosis in highly myopic individuals due to the normative database not representing all patient populations.

In addition to correcting for ocular magnification due to AL elongation via its built-in software, the RS-3000 Advance SD-OCT (Nidek, Co. Ltd. Japan) comprises two sets of normative databases embedded: the regular nonmyopic database for eyes with ALs < 26 mm and the long AL database for eyes with ALs between 26 and 29 mm^17,19^. The purpose of this study was to compare the diagnostic ability of these two distinct databases in the RS-3000 Advance SD-OCT device to accurately diagnose glaucoma in Taiwanese eyes with high myopia.

## Materials and methods

### Study population

The study was an observational cross-sectional study. Patients with high myopia (AL ≥ 26.0 mm) examined at the Glaucoma Service at the Department of Ophthalmology, Chang Gung Memorial Hospital, Taiwan, between January 2015 and August 2020 were included. The study was approved by the Institutional Review Board and the Ethics Committee of Chang Gung Memorial Hospital, Taiwan. All procedures adhered to the tenets of the Declaration of Helsinki. A written or signed informed consent was obtained for every participant in the study after providing adequate information and opportunity for the subject to consider all options, have all their questions answered, as well as continuing to response to the participants as the situation requires.

### Study protocol

Standard study protocols detailed below were compatible with methodology previously published by our group^20^. Comprehensive ophthalmic evaluation was performed for every patient as follows: slit-lamp examination, intraocular pressure measurements via Goldmann applanation tonometry, central corneal thickness measurements, gonioscopic examination by a Goldmann three-mirror lens, optic nerve head and fundus evaluation, digital color fundus photography (Digital Non-Mydriatic Retinal Camera, Canon, Tokyo, Japan), AL measurements by Optical Biometer AL-Scan (Nidek, Co. Ltd. Japan), central 30–2 Swedish Interactive Threshold Algorithm standard automated perimetry using a Humphrey Field Analyzer (Carl Zeiss Meditec, Dublin, CA, USA), measurements of the best-corrected visual acuity (BCVA), automatic objective determination of the refractive errors and examinations using the RS-3000 Advance SD-OCT (Nidek, Co. Ltd. Japan).

The inclusion criteria comprised a BCVA ≥ 20/20 in Snellen equivalents, an AL ≥ 26.0 mm, a normal anterior segment, open angle by gonioscopy, the presence of stereoscopic color fundus photographs for both eyes, visual field (VF) findings within three months of the SD-OCT exam, and the presence of normal or glaucomatous VF defects as determined by the Humphrey Field Analyzer. Exclusion criteria comprised previous refractive or intraocular surgery, vitreoretinal diseases affecting retinal thicknesses such as that of the epiretinal membrane, poorly controlled hypertension, neurologic diseases that might cause RNFL damage or VF defects, diabetes mellitus, degenerative myopia with patchy chorioretinal atrophy or choroidal neovascularization and low-quality SD-OCT images (signal strength index < 6/10). Standard study protocol.

### Glaucoma diagnosis

Glaucomatous optic neuropathy (GON) was diagnosed if the optic disc had a glaucomatous appearance, which was defined as having localized or diffuse neurorim thinning of the optic nerve head and/or RNFL defects corresponding to glaucomatous VF defects. By definition, glaucomatous VF defects refer to those meeting one or more of the following criteria under standard automated perimetry results: (1) a cluster of three points with probabilities of < 5% on the pattern deviation map in at least one hemifield, including one point or more with a probability of < 1%, or a cluster of two points with a probability of < 1%; (2) glaucomatous hemifield test results outside the normal limits; and (3) a pattern standard deviation (PSD) beyond 95% of normal limits as confirmed by at least two reliable examinations (false positive/negatives < 15%, fixation losses < 15%).

Eyes were assigned to the glaucomatous group if they were diagnosed with GON and had corresponding glaucomatous VF defects. Eyes were assigned to the normal group if they did not have glaucomatous appearances of the optic nerve head, visible RNFL defects, or glaucomatous VF defects on two reliable standard achromatic perimetry (SAP) examinations. Eyes with GON but without glaucomatous VF defects, a condition also known as preperimetric glaucoma, were excluded.

To ensure accuracy and consistency of our outcomes, two masked, experienced glaucoma specialists (HSLC and DWL) reviewed the diagnosis of each eye included. If there were discrepancies in the diagnosis, a third reviewer (WWS) was brought in to decide the final diagnosis by voting.

### SD-OCT measurement

A high-resolution scan procedure of the RS-3000 Advance SD-OCT was used to obtain images of the mGCCs of all included participants. In terms of wide-area three-dimensional imaging of the posterior pole, OCT scanning over a 30° × 30° square area (equivalent to a 9 × 9 mm square area in the Gullstrand model eye) with scan densities of 512 A-scans vertically × 128 B-scans horizontally was performed.

Image quality was reviewed in detail, and only scans with a signal strength index > 6/10 and devoid of any artifacts were used for analysis. Any image with motion artifacts or incorrect segmentation was excluded from the analysis. The mGCC thickness was calculated with the software Navis-EX version 1.4.1 (Nidek, Gamagori, Japan), a combined viewing software that enables data from various Nidek diagnostic imaging devices to be stored and processed in a centralized database. This program also corrects for the effect of AL-related ocular magnification using a modified Littmann’s formula (Bennett’s formula)^21^. Upon correction, the mGCC thickness and significance maps were determined for a 9-mm-diameter circle, which was centered on the fovea.

**Figure 1:**
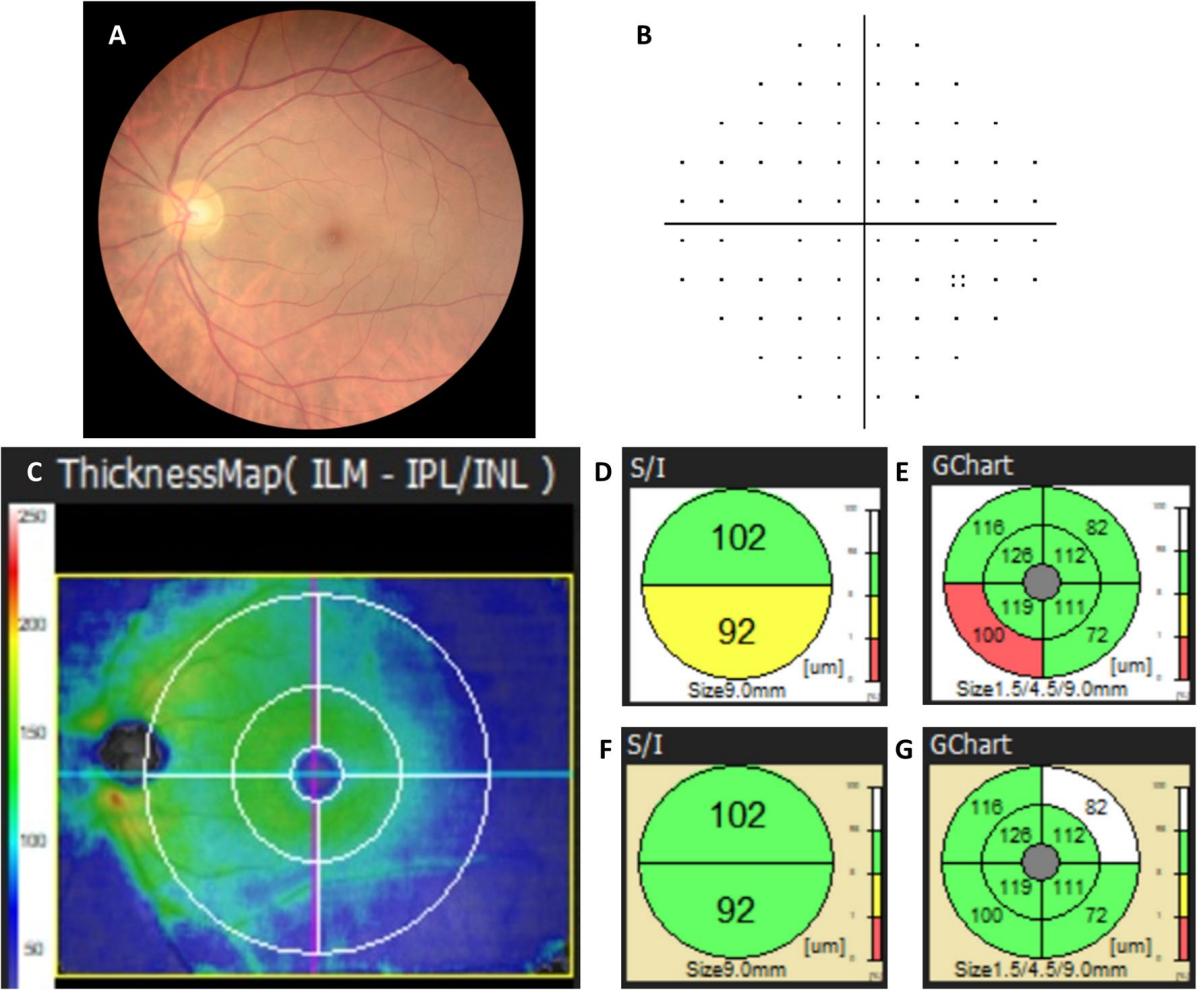
Fundus photography, automated visual fields, and spectral-domain optical coherence tomography images of a 40-year-old highly myopic female without glaucoma. The AL of the eye is 26.44 mm, and the refractive error (spherical equivalent) is − 7.5 D. (**A**) Color fundus photograph. (**B**) Pattern deviation of the Humphrey perimeter showing a relatively normal visual field. (**C**) A 9 mm × 9 mm square area of the RS-3000 Advance OCT macular ganglion cell complex thickness map (Nidek, Gamagori, Aichi, Japan) is shown. Thickness map pattern of the mGCC revealed suspect thinning area along the inferotemporal quadrant. Two types of circular maps are generated when using the regular normative database: (**D**) Superior/inferior (S/I) semicircle map and (**E**) GChart (8-sectored map). The second pair of circular maps, (**F**, **G**), were generated when using the long axial database. The thickness of each sector is not changed, whereas the color coding of the S/I and GChart mapping are changed after switching to the long axial database.

**Figure 2:**
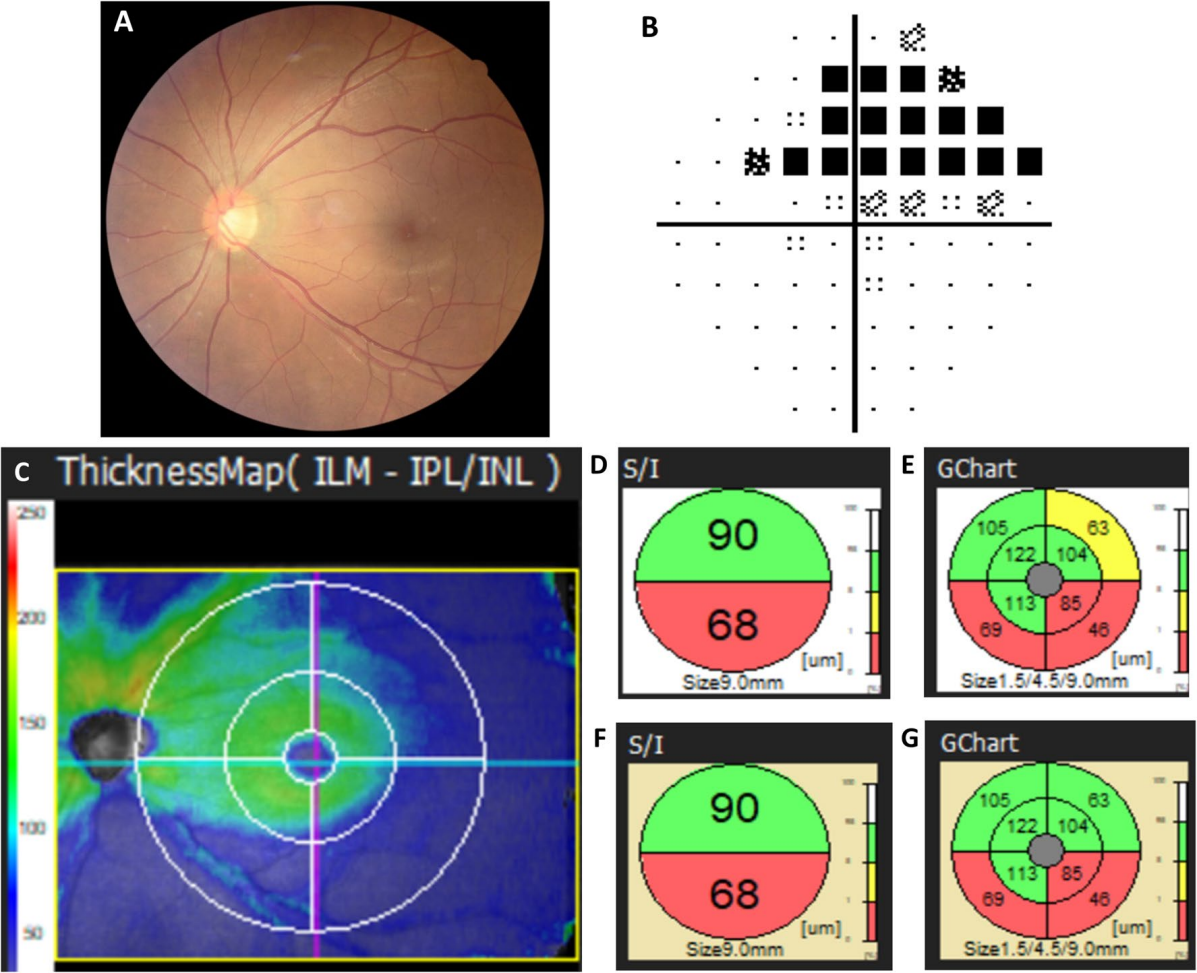
Fundus photography, automated visual fields, and spectral-domain optical coherence tomography images of a 56-year-old highly myopic male diagnosed with glaucoma. The AL of the eye is 26.17 mm, and the refractive error (spherical equivalent) is − 8.0 D. (**A**) Color fundus photograph showing an inferotemporal retinal nerve fiber layer defect. (**B**) Pattern deviation of the Humphrey perimeter showing a corresponding superior arcuate defect. (**C**) The mGCC thicknesses map revealed a prominent thinner inferior arcuate shape area compared with superior area. (**D**) Superior/inferior (S/I) semicircle map and (**E**) GChart (8-sectored map) when using the regular normative database. (**F**, **G**) The mGCC S/I and GChart circular maps corrected by the long axial length normative database, and color coding of the superotemporal sectors is changed after switching the database, however, color coding of the inferior mGCC thickness are not changed, which corresponds with the superior glaucomatous perimetric defect.

The mGCC thickness was measured from the internal limiting membrane (ILM) to the outer inner plexiform layer (IPL) based on OCT segmentation. The thickness and significance maps of the mGCC were categorized into two mapping types: a superior/inferior (S/I) semicircle map and an eight-sector map known as GChart (Figs. 1 and 2). A three-level color coding based on comparison with the built-in normative database was used in thickness comparisons: green (5–95% within the normal range), yellow (1–5% probability of being in the normal range), and red (< 1% probability of being within the normal range). Abnormal thinning is considered when there is at least one sector with red color coding in the mGCC thickness analysis. In the event that only yellow sectors are appreciated without red sectors, the patient is repeatedly analyzed as separately assumed *normal* and *abnormal* eyes in post-hoc analysis, so as to prevent erroneous estimation of diagnostic performance indices and to ensure robustness of the data.

The RS-3000 Advance SD-OCT system consists of two sets of normative databases: the regular and long AL databases (Figs. 1 and 2). The regular database was constructed from healthy Asians and Caucasians, with average ALs of 24.0 ± 0.9 mm and 23.4 ± 1.0 mm, respectively, and average refractive errors of − 1.0 ± 1.8 D and − 0.6 ± 1.7 D, respectively^17^. The long AL database consisted of data obtained from healthy Asian eyes with ALs ≥ 26.0 mm; the average AL was 27.1 ± 0.8 mm, and the average refractive error was − 8.1 ± 2.4 D^19^. The database consisted of retinal thickness distribution obtained from measuring the macula in three dimension.

All SD-OCT images were evaluated by two masked investigators (XCL and HSLC). In the event of disagreement between the two investigators, the image was examined again jointly with another senior investigator (DWL). A final decision was then attained for such images.

### Statistical analysis

All statistical analyses were performed using SPSS Version 26.0 (IBM Corp., NY, USA). Baseline characteristics and differences in the demographic features between the highly myopic normal group and highly myopic glaucomatous group were compared for statistical significance using Fisher’s exact test for dichotomous data or the two-sample t test for continuous data. To evaluate the clinical utility of each database in differentiating highly myopic normal and glaucomatous eyes based on mGCC thickness, the sensitivity, specificity, diagnostic accuracy and likelihood ratios of SD-OCT images were calculated. The mGCC scans were classified as abnormal thinning if there was at least one sector of the S/I or GChart significant maps < 1% (red color coding). In addition, the area under the receiver operating characteristic curve (AUROC) was calculated to assess the ability of mGCC thickness to discriminate glaucomatous eyes from normal eyes. Statistical significance was defined as p < 0.05.

### Ethical approval

All procedures performed in studies involving human participants were in accordance with the ethical standards of the Chang Gung Memorial Hospital Institutional Review Board (IRB) and with the 1964 Helsinki declaration and its later amendments or comparable ethical standards.

## Results

### Study population

Two hundred eighty-seven eyes from 151 subjects with high myopia were studied: 185 were primary open angle glaucomatous eyes (96 subjects), and 102 were normal nonglaucomatous eyes (55 subjects). Baseline demographics and perimetry parameters (mean deviation (MD) and PSD) of the two groups are shown in Table 1. There were no significant differences in sex, age, AL, intraocular pressure, central corneal thickness or refractive error between the groups.

**Table 1:** Demographics and ocular characteristics of the study population.

	Glaucoma	Normal	P values
Participants (no. of eyes)	96 (185)	55 (102)	
Male, n (%)	26	14	P = 0.77
Age (y/o)	47.3 ± 11.9	46.7 ± 13.6	P = 0.51
Axial length (mm)	27.28 ± 1.08	27.32 ± 1.25	P = 0.17
Refractive error (diopters)	− 7.81 ± 3.81	− 6.93 ± 4.15	P = 0.07
Central corneal thickness (µm)	544.4 ± 40.9	551.5 ± 42.9	P = 0.17
Intraocular pressure (mmHg)	17.6 ± 4.9	16.5 ± 4.4	P = 0.06
**Standard achromatic perimetry**			
MD (dB)	− 2.52 ± 1.40	− 1.24 ± 1.06	P < 0.001*
PSD (dB)	5.89 ± 4.17	2.65 ± 1.74	P < 0.001*

Data are presented as mean ± standard deviation.

*MD* mean deviation, *PSD* pattern standard deviation.

*Statistical significance shown at p < 0.05.

The average AL was 27.28 ± 1.08 mm in the glaucoma group and 27.32 ± 1.25 mm in the normal group (p = 0.17). All included eyes were phakic, with a mean refractive error of − 7.81 ± 3.81 D in the glaucoma group and − 6.93 ± 4.15 D in the normal group (p = 0.07). The MD and PSD values of SAP were significantly different between the groups (p < 0.001; Table 1).

### Analysis parameters of mGCC thickness

Tables 2 and 3 show the comparisons of mGCC thickness parameters for the highly myopic glaucoma group and normal group in the regular and long AL databases, respectively. The values were corrected with regard to AL-related magnification in the 9-mm-diameter circle. In both databases, the mGCC thicknesses of the glaucoma group were significantly thinner than those of the normal group in all sectors for S/I and GChart maps, except for the outer (superior, inferior, nasal and temporal quadrants) sectors.

**Table 2:** Comparison of the analysis chart parameters of mGCC thickness between highly myopic glaucoma group and highly myopic normal group (long axial database).

Total (µm)	Long axial database		P value	AROC (95% CI)
	Glaucoma (n = 185)	Normal (n = 102)		
**S/I**				
Superior	82.66 ± 13.50	94.03 ± 9.48	< 0.001*	0.773 (0.717–0.829)
Inferior	77.18 ± 16.27	93.21 ± 11.12	< 0.001*	0.800 (0.747–0.853)
**GChart**				
Inner TS	101.18 ± 18.70	112.69 ± 12.29	< 0.001*	0.692 (0.631–0.753)
Inner TI	92.95 ± 21.30	110.14 ± 15.94	< 0.001*	0.758 (0.701–0.816)
Inner NS	101.59 ± 20.06	113.55 ± 12.68	< 0.001*	0.688 (0.626–0.749)
Inner NI	93.51 ± 22.46	109.82 ± 17.14	< 0.001*	0.731 (0.671–0.791)
Outer TS	76.28 ± 19.14	87.27 ± 19.32	0.573	0.669 (0.606–0.733)
Outer TI	72.17 ± 22.02	87.18 ± 21.08	0.704	0.707 (0.648–0.767)
Outer NS	79.65 ± 22.02	102.59 ± 115.68	0.125	0.659 (0.595–0.723)
Outer NI	73.45 ± 22.69	90.85 ± 23.35	0.060	0.720 (0.660–0.780)

*AROC* Covariate-Adjusted Receiver Operating Characteristic, *CI* Confidence Interval, *S/I* Superior/Inferior, *TS* Temporal-Superior, *TI* Temporal-Inferior, *NS* Nasal-Superior, *NI* Nasal-Inferior.

*Statistical significance shown at p < 0.05.

**Table 3:** Comparison of the analysis chart parameters of mGCC thickness between highly myopic glaucoma group and highly myopic normal group (REGULAR database).

Total (µm)	Normative database		P value	AROC (95% CI)
	Glaucoma (n = 185)	Normal (n = 102)		
**S/I**				
Superior	80.30 ± 13.24	91.71 ± 9.53	< 0.001*	0.768 (0.711–0.824)
Inferior	75.15 ± 15.76	90.33 ± 10.45	< 0.001*	0.815 (0.763–0.867)
**GChart**				
Inner TS	98.83 ± 18.22	110.59 ± 13.67	0.001*	0.692 (0.630–0.753)
Inner TI	89.78 ± 20.89	107.38 ± 16.33	< 0.01*	0.756 (0.698–0.813)
Inner NS	99.13 ± 19.92	111.08 ± 13.87	< 0.01*	0.682 (0.620–0.743)
Inner NI	90.53 ± 20.93	106.88 ± 16.94	< 0.01*	0.732 (0.673–0.792)
Outer TS	73.28 ± 19.93	84.43 ± 20.68	0.13	0.665 (0.601–0.729)
Outer TI	70.04 ± 21.98	83.99 ± 21.33	0.47	0.693 (0.632–0.754)
Outer NS	77.44 ± 22.83	89.18 ± 22.56	0.43	0.655 (0.591–0.719)
Outer NI	71.71 ± 23.39	87.51 ± 23.59	0.06	0.708 (0.648–0.769)

*AROC* Covariate-Adjusted Receiver Operating Characteristic, *CI* Confidence Interval, *S/I* Superior/Inferior, *TS* Temporal-Superior, *TI* Temporal-Inferior, *NS* Nasal-Superior, *NI* Nasal-Inferior.

*Statistical significance shown at p < 0.05.

### Diagnostic abilities of the two normative databases

#### S/I maps

The diagnostic ability of S/I map in differentiating highly myopic glaucomatous eyes from highly myopic normal eyes using the regular and long AL normative databases was evaluated. Table 4 shows that in terms of the application of the regular normative database in S/I map evaluation, the sensitivity and specificity were 0.859 (95% CI 0.801–0.906) and 0.735 (95% CI 0.639–0.818), respectively. Although the sensitivity increased nonsignificantly to 0.860 (95% CI 0.801–0.906) using the long AL database, the specificity increased significantly to 0.882 (95% CI 0.804–0.938; p = 0.02) (Table 5).

**Table 4:** Discriminating ability of the regular normative database on macular ganglion cell complex thickness for glaucoma detection.

	Glaucoma (n = 185)	Normal (n = 102)
**Regular normative database**		
Superior/inferior		
Abnormal thinning	159	27
Within normal range	26	75
Sensitivity (95% CI)	0.859 (0.801–0.906)	
Specificity (95% CI)	0.735 (0.639–0.818)	
Positive likelihood ratio	3.25 (2.34–4.51)	
Negative likelihood ratio	0.19 (0.13–0.28)	
Positive predictive value	0.855 (0.809–0.891)	
Negative predictive value	0.743 (0.665–0.808)	
Accuracy	0.815 (0.766–0.859)	
**Regular normative database**		
GChart		
Abnormal thinning	169	26
Within normal range	16	76
Sensitivity (95% CI)	0.914 (0.863–0.950)	
Specificity (95% CI)	0.745 (0.649–0.826)	
Positive likelihood ratio	3.58 (2.56–5.01)	
Negative likelihood ratio	0.12 (0.07–0.19)	
Positive predictive value	0.867 (0.823–0.901)	
Negative predictive value	0.826 (0.746–0.885)	
Accuracy	0.854 (0.807–0.893)	

*CI* Confidence Interval.

**Table 5:** Discriminating ability of the long axial database on macular ganglion cell complex thickness for glaucoma detection.

	Glaucoma (n = 185)	Normal (n = 102)	p values (comparison between long axial and normal database)
**Long database**			
Superior/inferior			
Abnormal thinning	159	12	
Within normal range	26	90	
Sensitivity (95% CI)	0.860 (0.801–0.906)		p = 0.88
Specificity (95% CI)	0.882 (0.804–0.938)		p = 0.02**
Positive likelihood ratio	7.31 (4.28–12.47)		
Negative likelihood ratio	0.16 (0.11–0.23)		
Positive predictive value	0.930 (0.886–0.958)		
Negative predictive value	0.776 (0.707–0.833)		
Accuracy	0.868 (0.823–0.905)		
**Long normative database**			
GChart			
Abnormal thinning	165	15	
Within normal range	20	87	
Sensitivity (95% CI)	0.892 (0.838–0.933)		p = 0.61
Specificity (95% CI)	0.853 (0.769–0.915)		p = 0.046**
Positive likelihood ratio	6.06 (3.79–9.70)		
Negative likelihood ratio	0.13 (0.08–0.19)		
Positive predictive value	0.917 (0.873–0.946)		
Negative predictive value	0.813 (0.741–0.869)		
Accuracy	0.878 (0.835–0.914)		

*CI* Confidence Interval.

**Statistical significance shown at p < 0.05.

The estimation of the positive and negative likelihood ratios for the S/I map using the regular database were 3.58 (95% CI 2.56–5.01) and 0.12 (95% CI 0.07–0.19), respectively, and both values increased to 7.31 (95% CI 4.28–12.47) and 0.16 (95% CI 0.11–0.23) after the long AL database was applied. The diagnostic accuracy of S/I maps using the long AL database improved to 0.868 (95% CI 0.823–0.905) from 0.815 (95% CI 0.766–0.859) when using the regular database.

### GChart maps

The results of the evaluations of the Gchart significance maps for distinguishing highly myopic glaucomatous eyes from highly myopic normal eyes are shown in Tables 4 and 5. The sensitivity and specificity of GChart maps using the regular database were 0.914 (95% CI 0.863–0.950) and 0.745 (95% CI 0.649–0.826), respectively. When the long AL database was utilized, the sensitivity decreased nonsignificantly to 0.892 (95% CI 0.838–0.933), but the specificity improved significantly to 0.853 (95% CI 0.769–0.915; p = 0.046) (Table 5).

The estimation of the positive and negative likelihood ratios for the GChart using the regular database were 3.58 (95% CI 2.56–5.01) and 0.12 (95% CI 0.07–0.19), respectively, and both values increased to 6.06 (95% CI 3.79–9.70) and 0.13 (95% CI 0.08–0.19) after the long AL database was applied. The diagnostic accuracy of the GChart maps was 0.854 (95% CI 0.807–0.893) when the regular database was used, but it increased to 0.878 (95% CI 0.835–0.914) when the long AL database was applied.

To account for possible overestimation of diagnostic performance indices, a separate analysis was performed under the assumption that eyes with only yellow sectors were also classified as *abnormal*. Under this assumption, the Gchart maps provided an increased sensitivity of 0.910 (95% CI 0.899–0.931) but lowered specificity of 0.820 (95% CI 0.754–0.887) in the long AL database. Comparatively, there was no significant difference in both sensitivities and specificities between the analyses. Thus, the conclusion remained robust in both assumptions.

### Color coding changes after using the long AL database

There were 19 (10.3%) glaucomatous eyes and 38 (37.3%) normal eyes for which S/I mapping changed from abnormal thinning to within the normal range after the long AL database was applied (Table 6). The difference was statistically significant (p < 0.001). No eye showed S/I color coding changes from normal to abnormal when the long AL database was used.

**Table 6:** Color coding changes in glaucomatous and normal eyes after using long axial length database.

	Eyes with color coding changes, n (%)		p value
	Glaucoma (n = 185)	Normal (n = 102)	
**Superior/inferior**			
Changes from abnormal to normal	19 (10.3)	38 (37.3)	< 0.001*
Changes from normal to abnormal	0	0	N/A
**G chart**			
Changes from abnormal to normal	21 (11.4)	49 (48.0)	< 0.001*
Changes from normal to abnormal	0	0	N/A

*CI* Confidence Interval, *N/A* Not Applicable.

*Statistical significance shown at p < 0.05.

In terms of GChart mapping, 21 (11.4%) glaucomatous eyes and 49 (48.0%) normal eyes showed changes from abnormal thinning to within the normal range using the long AL database. The difference was statistically significant (p < 0.001). There were no eyes with changes from normal to abnormal thinning in the GChart of the long AL database.

## Discussion

Based on our results, the specificity and diagnostic accuracy of mGCC thickness for distinguishing glaucomatous eyes from normal eyes among highly myopic eyes were significantly improved using the long AL database. The increased specificity indicated that clinicians could utilize a highly myopic eye AL database to effectively decrease the number of false-positive diagnoses or to correctly identify highly myopic normal eyes misdiagnosed as glaucomatous eyes. Diagnostic sensitivity was similar for the S/I map but decreased for GChart mapping when using the long AL database.

Our results revealed that adjusting normative mGCC thickness data for long AL or highly myopic patients would provide better OCT specificity for glaucoma detection. Differentiating between glaucoma and high myopia has always been challenging. Structurally, ocular elongation and tilting of optic nerve head, two of the characteristic features of high myopia, may lead to visual field defects which resemble glaucoma^22,23^. Longitudinal follow-up of highly myopic patients may be required for accurate diagnosis of glaucoma. However, the role of OCT has become increasingly important as an adjunctive tool to help diagnosing glaucoma in myopic eyes^24,25^. Thus, OCT imaging devices containing normal high myopia databases with high diagnostic ability or accuracy for detecting glaucoma clinically in highly myopic individuals are optional considerations.

In a study by Nakanishi et al. using a long AL database, S/I maps were shown to have lower sensitivity and higher specificity than GChart maps^26^. Our study revealed similar results. The higher rate of false negatives in S/I maps compared to GChart maps could be due to the thinning focal area of the mGCC averaged over a wider normal mGCC thickness (S/I map)^26^. Another study by Nakanishi et al. investigating the effect of AL on mGCC thickness showed that despite correction for AL-associated magnification, the glaucoma diagnostic accuracy using mGCC in highly myopic eyes did not improve with the built-in regular normative database^27^. Overall, the trend of increased specificity of glaucoma diagnosis in highly myopic eyes was consistent with our findings, despite absolute value differences due to different population and number of eyes included.

In terms of color coding change, we showed that there were significant proportion changes in both S/I and GChart maps (37.3% and 48.0%, respectively) from abnormal to normal in the myopic normal eye group when using a highly myopic normative database. The significantly larger proportion of adjusted change (from red to yellow or green) in myopic normal patients as compared to the myopic glaucoma patients suggested that the use of high myopia database may help in correctly differentiating normal myopic from glaucomatous myopic eyes. Correspondingly, Seol et al. has revealed that via the implementation of a myopic normative database in using OCT color probability codes, the glaucoma diagnostic ability for OCT in myopic eyes was significantly improved^28^.

While this current study did not evaluate the diagnostic performance of RNFL thickness in highly myopic eyes using a highly myopic normative database, our results in mGCC thickness evaluation were consistent with the results of several reports investigating the former^29,30^. Biswas et al. showed that the myopic normative database had a higher specificity (63–100%) than the conventional normative database (8.7–87.0%) in the detection of RNFL abnormalities in highly myopic eyes^29^. Kim et al. revealed that the rate of false-positive errors (p < 1%) for RNFL thickness was significantly higher in the high myopia group (62.8%) than in the mild-to-moderate myopia group using a regular normative database^30^.

The limitations of our study include its observational, retrospective design. A previous study performed by our group had the limitation of a relatively small sample size^20^. However, this limitation was improved upon in this study due to its relatively large sample size. To prevent collinearity between eyes of the same patient from undermining the statistical power of our analysis, we have tested for multicollinearity with variance inflation factor (VIF), which was 1.51 between two eyes, representing collinearity not significant to affect the conclusion. Retrospective studies involving diagnostic examination may commonly overestimate the performance of the exam. Thus, prospective, longitudinally designed studies with larger sample sizes may be needed. Another limitation is the non-inclusion of preperimetric glaucoma patients in the current study. This was performed to better define and compare between regular and highly myopic eyes, with respect to the use of different normative databases in glaucoma diagnosis.

In conclusion, the mGCC thickness of highly myopic eyes has significantly improved specificity in differentiating normal, highly myopic eyes from glaucomatous eyes when using the long AL database embedded in the RS-3000 SD-OCT instrument. These results may strengthen the use of a long AL normative database in other OCT instruments, thus providing a more accurate diagnosis of glaucoma in highly myopic eyes.

## Data Availability

As the datasets involved were formatted together with medical record numbers from the hospital database, the IRB stipulation prevented the datasets from being made publicly available. However, if the reader has any need for the datasets as reference, the datasets used and/or analysed during the current study are available from the corresponding author on reasonable request.
